# Retinal functional ultrasound imaging (rfUS) for assessing neurovascular alterations: a pilot study on a rat model of dementia

**DOI:** 10.1038/s41598-022-23366-8

**Published:** 2022-11-14

**Authors:** Clementine Morisset, Alexandre Dizeux, Benoit Larrat, Erwan Selingue, Herve Boutin, Serge Picaud, Jose-Alain Sahel, Nathalie Ialy-Radio, Sophie Pezet, Mickael Tanter, Thomas Deffieux

**Affiliations:** 1 Institute Physics for Medicine Paris, INSERM U1273, ESPCI PSL Paris, CNRS UMR 8631, PSL Research University, Paris, France; 2 NeuroSpin, Institut Des Sciences du Vivant Frédéric Joliot, Commissariat À L’Energie Atomique Et Aux Energies Alternatives (CEA), CNRS, Université Paris-Saclay, 91191 Gif-Sur-Yvette, France; 3 Faculty of Biology, Medicine and Health, School of Biological Sciences Division of Neuroscience and Experimental Psychology, University of Manchester, Manchester, M13 9PL UK; 4 Wolfson Molecular Imaging Centre, University of Manchester, 27 Palatine Road, Manchester, M20 3LJ UK; 5 Geoffrey Jefferson Brain Research Centre, Manchester Academic Health Science Centre, Northern Care Alliance and University of Manchester, Manchester, UK; 6 Institut de La Vision, Sorbonne Université, INSERM, CNRS, 17 Rue Moreau, 75012 Paris, France; 7 Department of Ophthalmology, The University of Pittsburgh School of Medicine, Pittsburgh, PA 15213 USA; 8 Department of Ophthalmology and Vitreo-Retinal Diseases, Fondation Ophtalmologique Rothschild, 75019 Paris, France

**Keywords:** Translational research, Acoustics, Imaging techniques, Neuro-vascular interactions, Retina

## Abstract

Fifty million people worldwide are affected by dementia, a heterogeneous neurodegenerative condition encompassing diseases such as Alzheimer’s, vascular dementia, and Parkinson’s. For them, cognitive decline is often the first marker of the pathology after irreversible brain damage has already occurred. Researchers now believe that structural and functional alterations of the brain vasculature could be early precursors of the diseases and are looking at how functional imaging could provide an early diagnosis years before irreversible clinical symptoms. In this preclinical pilot study, we proposed using functional ultrasound (fUS) on the retina to assess neurovascular alterations non-invasively, bypassing the skull limitation. We demonstrated for the first time the use of functional ultrasound in the retina and applied it to characterize the retinal hemodynamic response function in vivo in rats following a visual stimulus*.* We then demonstrated that retinal fUS could measure robust neurovascular coupling alterations between wild-type rats and TgF344-AD rat models of Alzheimer’s disease. We observed an average relative increase in blood volume of 21% in the WT versus 37% for the TG group (*p* = 0.019). As a portable, non-invasive and inexpensive technique, rfUS is a promising functional screening tool in clinics for dementia years before symptoms.

## Introduction

Dementia is a heterogeneous neurodegenerative pathology that yields neurological symptoms severe enough to interfere with daily life, such as loss of memory or thinking disabilities. Dementia encompasses Alzheimer’s disease (AD), vascular dementia (VaD), Lewy body dementia (LBD), frontotemporal dementia (FTD), and Parkinson’s disease (PD) and affects more than 50 million people worldwide. It is expected to grow three folds before 2050 and is quickly becoming a significant challenge, impacting families and healthcare systems significantly.

The diagnosis of neurodegenerative and cerebrovascular diseases has witnessed considerable advances in recent decades thanks to the development of increasingly high-performance imaging technologies such as Magnetic Resonance Imaging (MRI) or Computed Tomography (CT).

For example, anatomical MRI and CT are routinely used with a high success rate^1^ as a standard workup for patients with symptoms of dementia. However, by the time the disease is detected in those anatomical images, much tissue damage is already done, and any treatment is unlikely to improve the disease outcome drastically.

Beyond morphological scanning, functional brain imaging, i.e., imaging of the brain activation, for example, following an external stimulus, is envisioned to provide early biomarkers of those disorders and help improve treatment outcomes. For instance, early widespread alterations of functional connectivity have been shown in Alzheimer’s disease^3–7^. Recent neuroimaging studies in patients with Alzheimer’s have also shown that neurovascular coupling response impairment could also occur before neurodegenerative changes^8–12^. Assessing the neurovascular response to a stimulus could thus provide an early marker of the pathology^13^ years before the symptoms.

Unfortunately, functional imaging of the adult brain requires an fMRI scanner, a costly and complex system unsuitable as an early screening tool. Researchers have thus recently turned to the retina to propose alternative techniques to assess the neurovascular response. The retina develops from the same embryonic tissue as the brain and is made of several neuronal layers as the cortex. As it requires a constant supply of glucose and oxygen to function, neurovascular coupling is also present^14,15^ and may reflect widespread alterations. Moreover, the retina is much more accessible to optical imaging and can be used as a natural window on some neurological disorders as it is part of the central nervous system.

Optical techniques relying on the Doppler effect or dynamic fundus imaging were proposed to measure retinal blood flow increase following a light stimulation and are now evaluated in clinics, including in the context of AD^16,17^. Although those techniques show promising results, they only take measurements from larger surface vessels and rely on tiny changes in vessel diameters following tens of seconds of stimulation. Given its high potential as an early clinical biomarker of the brain’s health, there remains a need to explore the feasibility of different neurovascular assessment techniques.

In this work, we thus propose a new approach based on functional ultrasound imaging to measure the retinal hemodynamic response function in vivo with high sensitivity. Functional ultrasound (fUS) is a relatively recent functional neuroimaging technique analog to functional MRI that uses ultrafast plane waves ultrasound imaging to acquire images of the brain microvasculature at high spatiotemporal resolution^18,19^. It is highly sensitive and can detect tiny changes in blood volumes associated with local functional hyperemia, even in deep cerebral structures^20^. The technique has been applied successfully to image brain activity in different species and humans intra-operatively^21^ or neonates through the transfontanellar window^22^ as in adults, the skull bone acts as a partial barrier to ultrasonic propagation.

We first explored how to obtain robust light-induced functional activation maps in the rat retina using fUS. To do so, we investigated the rat retina microvascular architecture using ultrasound ultrafast localized microscopy (ULM) using contrast agents to help with the probe positioning.

Using repeated visual short stimuli (0.8 s), we characterized the hemodynamic response function (HRF) of the retina in wild-type rats to obtain robust hemodynamic response parameters.

Ultimately, our objective is to demonstrate our approach’s capability to assess the presence of neurovascular alterations non-invasively with ultrasound. To test this hypothesis, we compared the hemodynamic response function obtained by light stimulation in the TgF344-AD rat model of Alzheimer’s disease and wild-type animals.

## Material and methods

Methods were carried out following relevant guidelines and regulations and in compliance with ARRIVE guidelines^23^.

### Ethics statement

Animals were housed under controlled temperature (21 ± ten °C) and humidity (55 ± 10%), with a 12-h light/12-h dark cycle. Food and water were available ad libitum during all experiments. This study was approved by the local committee for animal care (*Comité d'éthique en matière d'expérimentation animale Paris Centre* No. 59) under the Agreement No. 24933.

### Animal preparation

In order to map the deep vascular anatomy of the retina, ULM imaging was performed on N = 1 male Sprague–Dawley rat weighing 400 g. rfUS imaging was performed on two groups of rats of 18 months of age: one group of TgF344-AD rats (TG group, n = 6) and another group of wild-type littermates (WT group, n = 6). For both types of imaging, animals were anesthetized intraperitoneally with a mixture of Ketamine (Imalgene^®^, 40 mg/kg) and Medetomidine (Domitor^®^, 0.3 mg/kg). After anesthesia induction, the animal was placed in a stereotaxic frame to stabilize its head. The anesthesia level was adjusted, if necessary, throughout the experiment. The body temperature was controlled by a rectal probe and maintained at 37 ± 0.5 °C by a feedback-controlled heating pad. Neomycin‐polymyxin B (Tevemyxine^®^ Collyre; TVM, Lempdes, France) and N-acetylcysteine (N.A.C.^®^ Collyre) ophthalmic solutions were instilled in the imaged eye (2 drops each) to prevent corneal ulceration after the imaging session. For rfUS imaging, one drop of tropicamide (Mydriaticum^®^ 0.5% Collyre) was instilled 30 min prior to the acquisition to induce mydriasis and cycloplegia. Tropicamide is an anticholinergic drug that produces short-acting mydriasis and cycloplegia and allows better retina examination. Anticholinergic agents competitively block the binding of the neurotransmitter acetylcholine at muscarinic receptors located on the eye’s ciliary body, inducing paralysis of the ciliary muscle that controls pupil dilation.

All animals were awakened with a subcutaneous injection of Atipamezole (Antisedan^®^, 0.1 mg/kg) at the end of the experiment.

### Retinal imaging

#### Ultrasound localization microscopy

**Figure 1:**
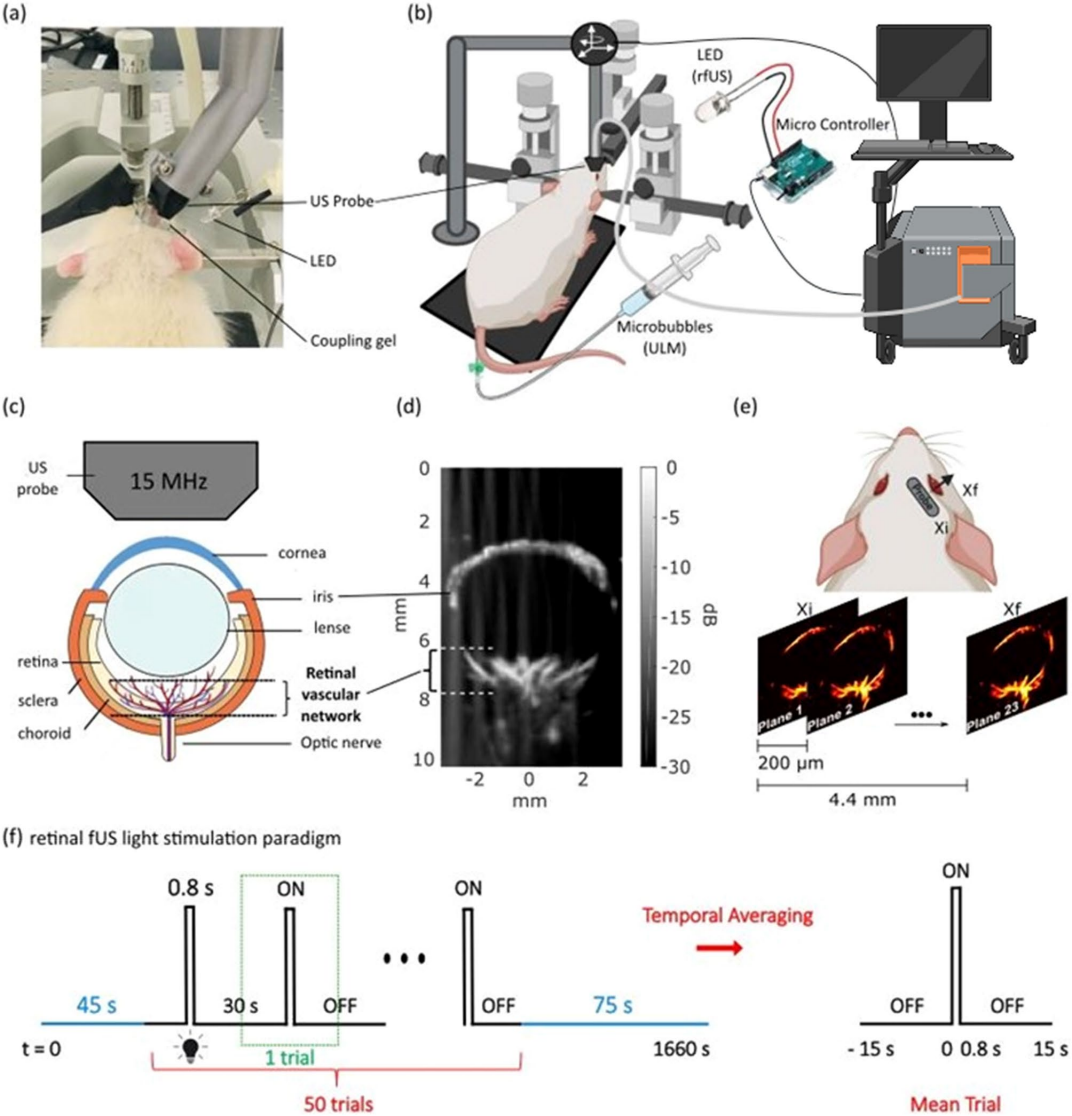
Ultrasound Localization Microscopy and fUS Imaging Set Up. (**a**) Picture of the experimental setup. The animal is anesthetized and placed in a stereotaxic frame for the correct positioning of the US probe. After eye drop and mydriatic instillation, the eye is covered with ophthalmic coupling gel. The probe is tilted to image the plane shown in (**d**). (**b**) Scheme of the entire setup. The probe is connected to a functional ultrasound scanner. The microbubbles solution is a contrast agent administered intravenously via one of the rat tail lateral veins for ULM. The LED delivers light flashes for rfUS imaging and is triggered by the scanner through a microcontroller (Arduino) for precise timings. (**c**) Scheme of the rat eye, its principal structures, and the imaging field comprising the retinal vascular network. (**d**) Doppler image of a rat eye. The retinal vascular network is visible. (**e**) ULM scanning details. The scan starts at the initial position *x*_*i*_ in 200 µm steps along the mediolateral axis to the final position *x*_*f*_. (**f**) Retinal fUS imaging stimulation design. The acquisition consists of a baseline recording of 45 s followed by 50 consecutive trials and terminated by a recovery period of 75 s, during which the RBV is continuously recorded. One trial consists of 15 s OFF, followed by a short light flash of 0.8 s ON and another 15 s OFF. One acquisition lasts 28 min. The mean trial is obtained via temporal averaging of the successive single trials. Figure created with BioRender.com.

ULM imaging was performed non-invasively through the eye using gas microbubbles, microbubbles are FDA-approved ultrasound contrast agents that can incidentally be used for super-resolution vascular imaging. The microbubbles (SonoVue™, Braco, Italy) were reconstituted in the supplied 5 mL of saline solution per vial (according to the manufacturer’s instructions) and administered via intravenous bolus injections of 200 µL in a lateral tail vein during the scan^24^ (Fig. 1a,b). Blocks of 400 ultrasound images obtained at a 1000 Hz framerate (each ultrasonic frame is a compound image acquired with transmissions at different compound angles − 6°, − 3°, 0°, + 3°, + 6° fired at a 5000 Hz Pulse Repetition Frequency, 8 V). The functional ultrasound scanner prototype (Iconeus One, Iconeus, Paris, France) drives a 15-MHz ultrasound probe with 128 elements (Fig. 1c). The scan consists of 23 successive imaging planes acquired every 0.2 mm over 4.4 mm from an initial position (Xi) over the nasal side of the eye to a final position (Xf) over the temporal side of the eye (Fig. 1d,e). We accumulated images for 180 s for each plane (900 compounded images). The entire 3D scan lasted 63 min. Density maps (D) were computed by counting all the positions detected in one pixel during the acquisitions. Velocity maps were computed as the mean velocity of every microbubble which passed through the pixel during the acquisition. For more details on the data processing, please refer to the data processing section described in Demene et al.^25^.

#### Experimental paradigm

All measurements were performed in the right eye according to the following schedule. After a short resting period to obtain stable hemodynamic conditions, power doppler measurements were performed. The acquisition consists of a baseline recording of 45 s followed by 50 consecutive trials and terminated by a recovery period of 75 s, during which the RBV is continuously recorded. Each trial comprises 15 s without stimulation, a short 0.8 s pulse of white light, and another 15 s without stimulation. One complete acquisition lasted 1660 s.

#### Light stimulation

The animal was maintained in a dim light room for 45 min before starting the imaging session. A custom-built device was used for light stimulation, consisting of a white LED delivering light flashes through a microcontroller (Arduino Uno, Ivrea, Italy). The microcontroller was programmed with the Arduino Software (IDE). The LED was directly triggered through the microcontroller to synchronize the stimulation pattern with the scanner. It was set to flash for 0.8 s every 30 s to complete 50 trials (Fig. 1f).

#### Functional ultrasound sequences

Functional ultrasound imaging acquisitions were performed non-invasively through the eye using the same probe for ULM imaging. The ultrasonic probe was placed upon the eye with acoustic coupling gel to image the retinal plane of interest where the long posterior ciliary arteries (LPCAs) were visible as they represent a reproducible and central landmark of the vasculature of the posterior part of the eye. The transducer was connected to a prototype functional ultrasound scanner (Iconeus One, Iconeus, Paris, France). Data was acquired by emitting a group of 11 plane waves tilted from − 10° to + 10°, fired at a 5.5 kHz pulse repetition frequency. The back-scattered echoes of each group were summed to get compound images at 500 Hz framerate, which allows correct sampling of the blood signal^19^. Doppler images were computed from blocks of 200 compound images averaged after filtering with the SVD Spatio-temporal clutter filter^26^ and removing the 60 first singular values to discard noise and tissue motion. Each pixel of the final Power Doppler image is reconstructed on a 100 × 100μm^2^ pixel in the plane, and the slice thickness is approximately 300 μm.

#### Activation map

Data preprocessing consisted of linear detrending, and neither spatial nor temporal smoothing was used. For each animal, initial activation maps were computed with a single-subject generalized linear model (GLM) approach routinely used for processing fMRI data^27^. The stimulus pattern was convolved by the default SPM canonical HRF^27^. Statistical parametric maps were generated for each session, including a z-score map, *p*-value map, stimulation map, and baseline map. Individual relative retinal blood volume (rRBV) maps were obtained from the GLM coefficients.

For display purposes, the activation maps were thresholded using the Bonferroni correction for multiple comparisons (uncorrected *p* < 0.05) and overlaid onto the corresponding grayscale mean Doppler image of the animal eye. The mean increase of relative retinal blood volume during activation was estimated from a region of interest (1.4 mm diameter) centered on the peak z-score of the activation map. Data processing and analysis were performed using Matlab (MATLAB Release 2018a, The MathWorks, Natick, Massachusetts, United States).

#### Hemodynamic response measurement and parameters estimation

The retinal hemodynamic response was extracted and averaged from the same region of interest, and the times series were averaged between trials for each animal. For a visual comparison, the hemodynamic response was averaged within groups for comparison between the transgenic retinal hemodynamic response and the control response.

In order to extract quantitative parameters, we fitted the hemodynamic response using a simple model. While several models of hemodynamic responses have been proposed in the fMRI literature, such as the two-gammas function or inverse logit function^28^, we choose to use a simpler parametrized model. This parametric model allows direct interpretation of key physiological parameters^29^ such as the initial response delay, the response rise time, the response amplitude, the response decay time, the undershoot amplitude, and return to baseline time. For this, we have opted to use the four cosines model, which is the concatenation and scaling of four half-period cosines and is determined by six independent parameters (see supplementary Fig. 1). Non-linear curve fitting of the RBV response by the four cosines model was then performed using the interior point method with constraints (Matlab 2018a, The MathWorks, Inc., Natick, Massachusetts, United States.).

In order to evaluate the number of trials necessary to obtain robust hemodynamic parameters, we performed a bootstrap analysis by randomly sampling with replacement a subset of trials in the experiment and rerunning the fit and parameters estimations one hundred times to obtain stable values. Curves representing the different parameters’ robustness were constructed by evaluating their standard deviation and mean.

### Statistical analysis

To compare the different hemodynamic response parameters of the hemodynamic retinal response of the transgenic (TG) and wild-type (WT) groups, a two-tailed Wilcoxon test was performed (because of TG group non-normal distribution) using GraphPad Prism (GraphPad Software, La Jolla California USA). To assess significance, we considered *p* < 0.05 statistically significant.

## Results

### Ultrasound localization microscopy reveals the full microvasculature of the rat eye in depth

**Figure 2:**
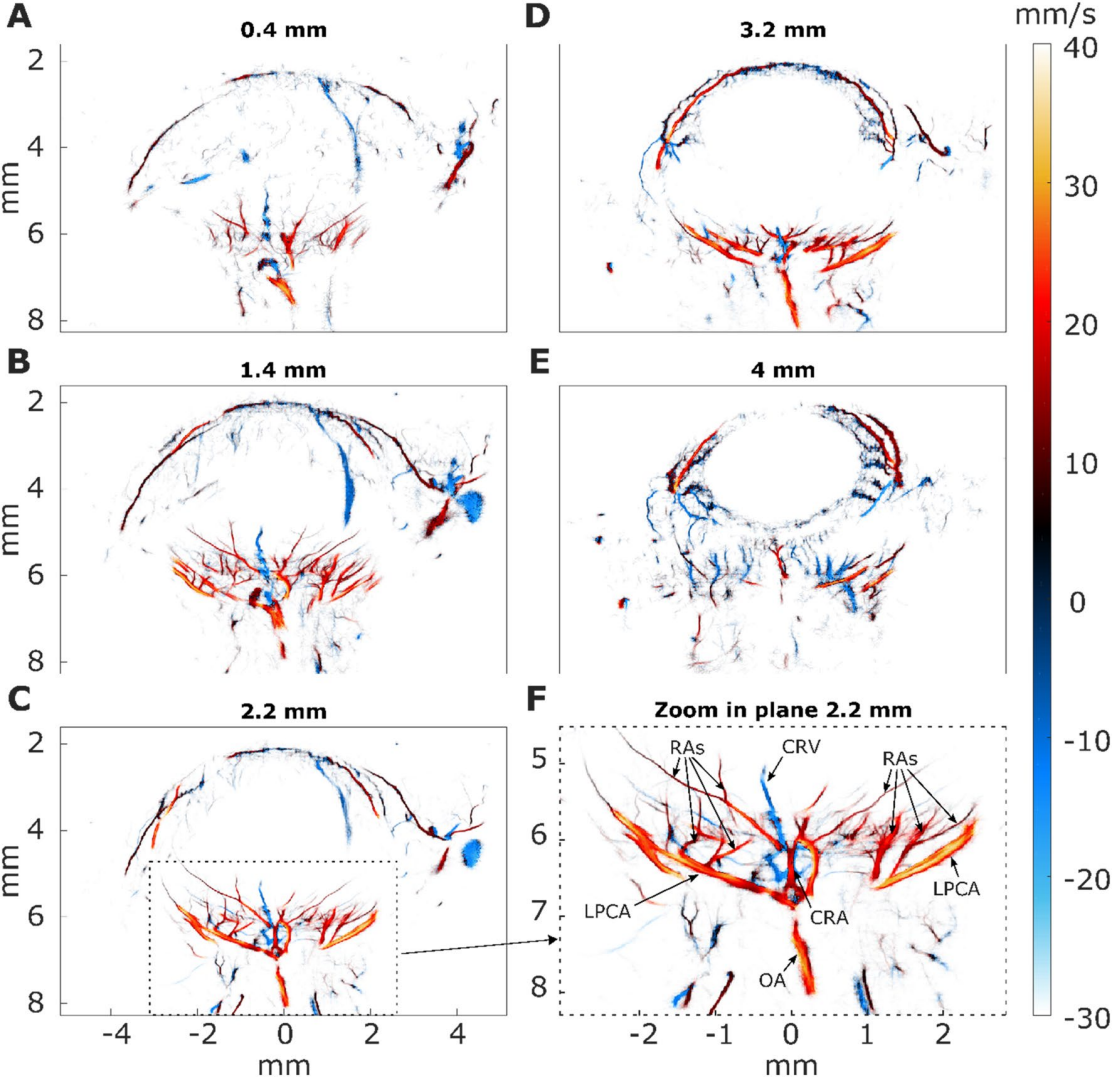
Super-resolution imaging reveals the vascular anatomy of the rat eye. Intravenous injection of microbubbles increases the resolution from approximately 100 microns to approximately 15 microns. Twenty-three planes were acquired every 200 µm in the anteroposterior direction. 5 out of the 23 ULM planes are displayed, (**A**) plane 10, (**B**) plane 15, (**C**) plane 17, (**D**) plane 20, (**E**) plane 23. (**F**) Zoom in the retinal microvasculature. The retina is perfused by the central retinal artery (CRA), which enters the retina at the optic disk, traveling through the optic canal alongside the optic nerve. The CRA and long posterior ciliary arteries (LPCAs) arise from the ophthalmic artery (OA), the first major branch of the internal carotid artery. Along its course, the CRA gives off radial arterioles (RAs) and smaller vessels on the vitreal surface of the retina. Blood returns through radial venules on the retinal surface that empty into the central retinal vein (CRV) in the optic nerve.

In order to better understand the probe positioning and orientation relative to the retina, we performed several Ultrasound Localization Microscopy images that covered the rat eye in different slices. ULM is based on microbubble injection in the blood and their tracking by ultrasound. It allows for reconstructing highly detailed vascularization maps of the retina, including quantitative flow velocities. Five different planes are shown in Fig. 2. Images were color-coded using the flow direction to allow the discrimination between veins (blue, away from the probe) and arterioles (red, towards the probe). Zoom in the retinal vascular bed from one of the five planes (Fig. 2C) is displayed in Fig. 2F.

The ophthalmic artery (OA), clearly visible at the bottom of the zoom image, originates from the internal carotid artery. In the OA, the blood flow velocity is about 25 mm/s. Immediately posterior to the globe, the OA bifurcates into a medial and a lateral long posterior ciliary artery (LPCA, between 30 and 40 mm/s) that run toward either side of the globe, and the central retinal artery (CRA, 20 mm/s) as it runs through the center of the optic nerve. The CRA emerges from the optic nerve within the globe to enter the retina at the level of the optic disc, where it branches into several major vessels giving off to radial arterioles (Ras) to supply the retinal vascular network. In first-order arterioles branching directly from the CRA, the blood flows at speeds up to 23 mm/s. As the order increases in subsequent ramifications, the speed decreases to 5 mm/s in the smallest arterioles detectable by ULM. The blood flows back through radial venules (RVs) that empty into the central retinal vein (CRV, ~ 10 mm/s) that runs adjacent to the CRA.

The CRA branches directly from the ophthalmic artery to pierce the medial aspect of the optic nerve sheath, approximately 10–15 mm behind the globe. The CRA runs adjacent to the central retinal vein (CRV) through the center of the optic nerve. It emerges from the optic nerve within the globe, where it branches into four significant vessels: the arteriola nasalis retinae superior and inferior, and the arteriola temporalis retinae superior and inferior.

### Retinal functional ultrasound can measure robust retinal activation during light stimulation

**Figure 3:**
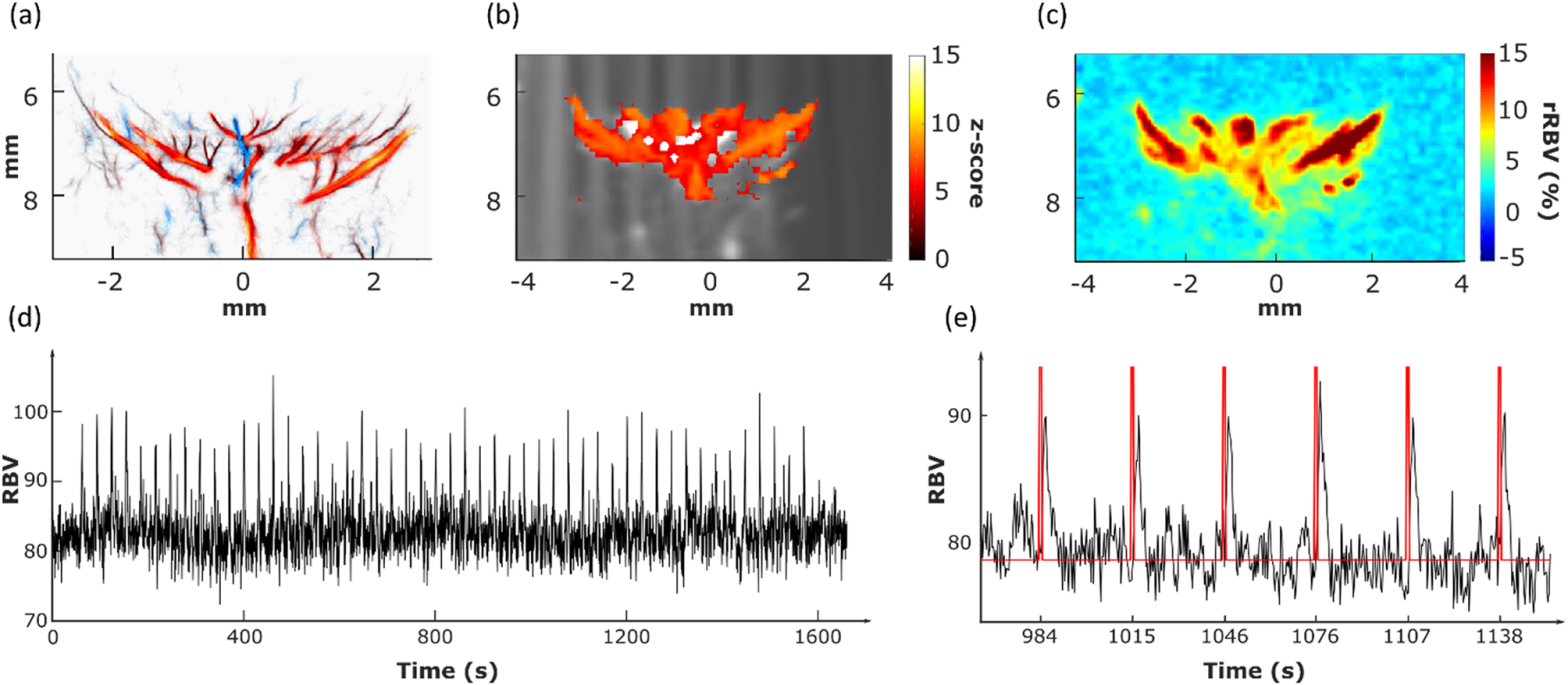
Neurovascular coupling in the Retinal Vascular Network (**a**) Doppler image of the rat eye. (**b**) Zscore activation map of the retina. (**c**) Delta RBV Map is showing activation percentage compared to baseline for each pixel. (**d**) Activation Map-based ROI (*p* < 0.05). (**e**) ROI Mean RBV temporal Signal over the total acquisition (1660 s) comprised 50 trials. Each peak is retinal hyperemia. (**f**) Microvasculature of the retina by ULM (**g**) Zoom in the RBV temporal Signal allows better visualization of single-trial responses. The visual stimulation pattern is represented in red. (**h**) Mean trial response over the 50 trials with SEM. The visual stimulation pattern is represented in red.

In all animals, the probe was manually positioned on the slice y = 2.2 mm, which allowed us to visualize the long posterior ciliary arteries (LPCAs) as they represent a reproducible and central landmark of the vasculature of the posterior part of the eye. Ultrafast Doppler imaging performed for functional ultrasound imaging provided the mapping of retinal blood volume (Fig. 3b RBV at baseline in grayscale) without requiring contrast agents (microbubbles). It gave access to blood volume measurements in the main arteries and arterioles (as compared with ULM, Fig. 3a). A zscore map was estimated by applying the Generalized Linear Method to the successive ultrafast Doppler images, as described in the method section, and showed strong activation of the retina (Fig. 3b). RBV increased by about 20% on average and up to 30% in the area during light stimulation (Fig. 3c). The peak of the zscore map was detected in the retinal bed around the long posterior ciliary arteries and used to automatically position the region of interest in the image (circle diameter of 1.4 mm).

An RBV profile was extracted using the newly defined region of interest. Individual stimulus trials are visible during the experiment with a high signal-to-noise ratio (Fig. 3d). A detailed zoom on a few trials allows us to investigate qualitatively further the hemodynamic response dynamics (Fig. 3e).

Retinal hemodynamics have been studied with different imaging techniques^30,31^ recently, including ultrafast ultrasound imaging^32^. To our knowledge, this is the first study of retinal neurovascular coupling by functional ultrasound imaging.

### Retinal hemodynamic response parameters estimation

We then wished to extract quantitative parameters of the hemodynamic response, as they could yield valuable information on possible alterations of the neurovascular coupling in clinics. We thus averaged the different trials of a single experiment and fitted the average response with the 4-cosines model.

We obtained quantitative values for the main hemodynamic response parameters in all (n = 6) control wild-type rats.

**Figure 4:**
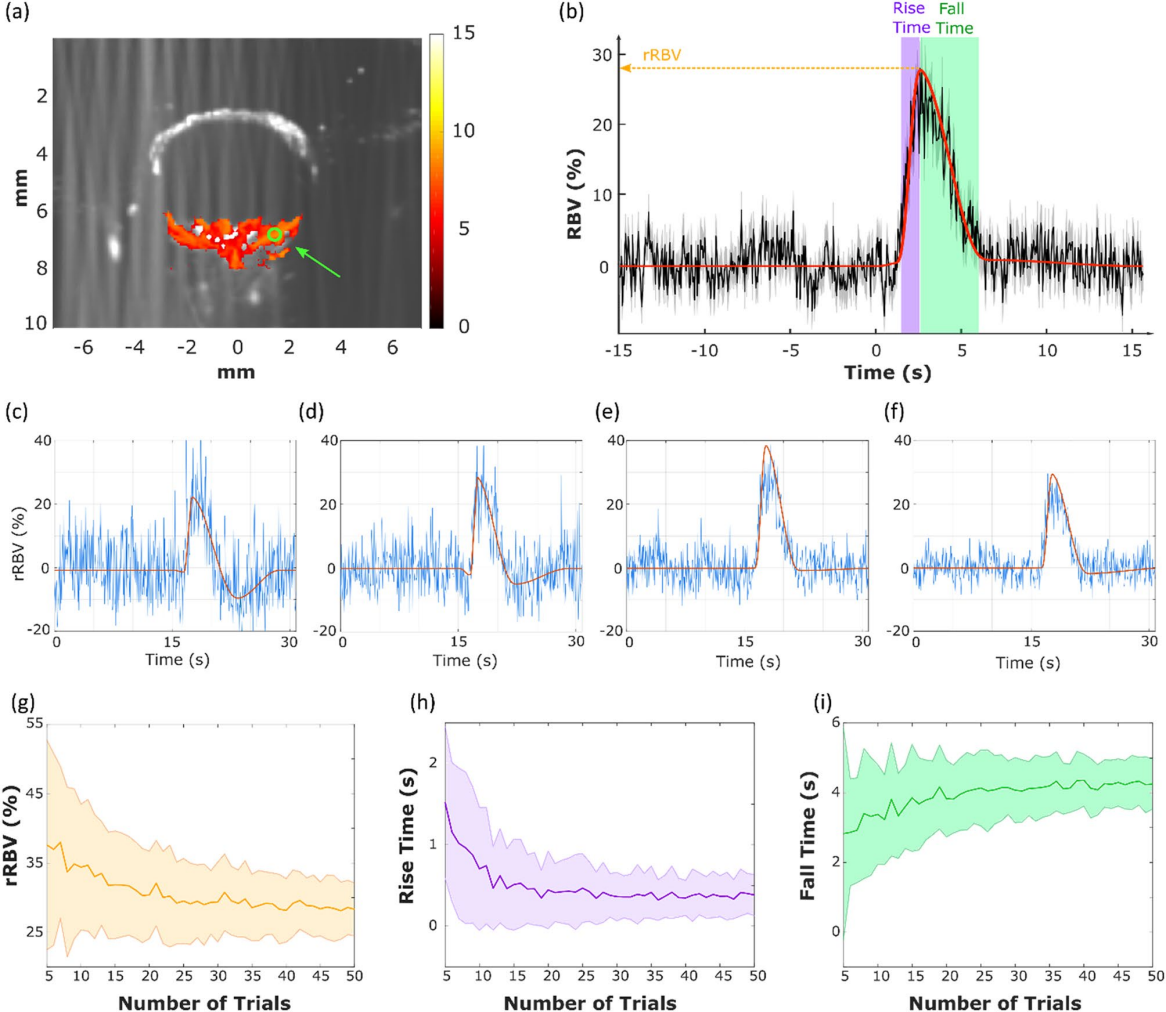
Robustness of the HRF Parameters*.* (**a**) Zscore activation map and automatic positioning of the ROI over the peak response (**b**) Average on all trials of the hemodynamic response (black curve) within the chosen ROI with its fitted hemodynamic response model (red curve). The relative amplitude, rise time, and fall time are extracted from the fitted hemodynamic response model (**c**) Examples of hemodynamic responses for four individual trials (blue curves) and their associated fitted model (red curves). (**g**)–(**i**) Evolution of the mean and standard deviation of the relative retinal blood volume, rise and fall times parameters depending on the number of stimuli performed, estimated using a bootstrap approach.

To evaluate the robustness of the parameters computed from the cosine fit and estimate the minimum number of trials N within a fUS acquisition necessary for a stable HRF extraction, we draw on the bootstrap inference method. The sample comprises 50 trials of a single acquisition, and each trial corresponds to a single visual stimulus. For each sample size N from 5 to 50, we computed 100 “bootstrap samples” of size N. The evolution of the parameters’ standard deviation is plotted as a function of the subset size, i.e., the number of trials using random sampling with replacement (Fig. 4).

The results show that the three primary parameters (amplitude response, rise time, and fall time) first present a significant bias when few trials are used in the estimation and then become stable (relative error < 5% of the final value) after 25 trials which correspond to roughly 12 min of acquisition (Fig. 4). The other parameters from the 4-cosines model (i.e., initial delay, relative undershoot amplitude, and back to baseline time, see supplementary Fig. 1) were difficult to estimate reliably and were not used in the subsequent analysis.

### Application to neurovascular coupling alterations: comparison with the Alzheimer rat model

To evaluate the rfUS strategy for detecting neurovascular coupling alterations, we compared the hemodynamic response parameters between control (n = 6) and TgF344-AD rats (n = 6). The measurements were made on animals aged 18 months. At this age, the neurodegenerative disease is known to have already progressed in the brain and eye, mimicking the human pathology^33,34^.

**Figure 5:**
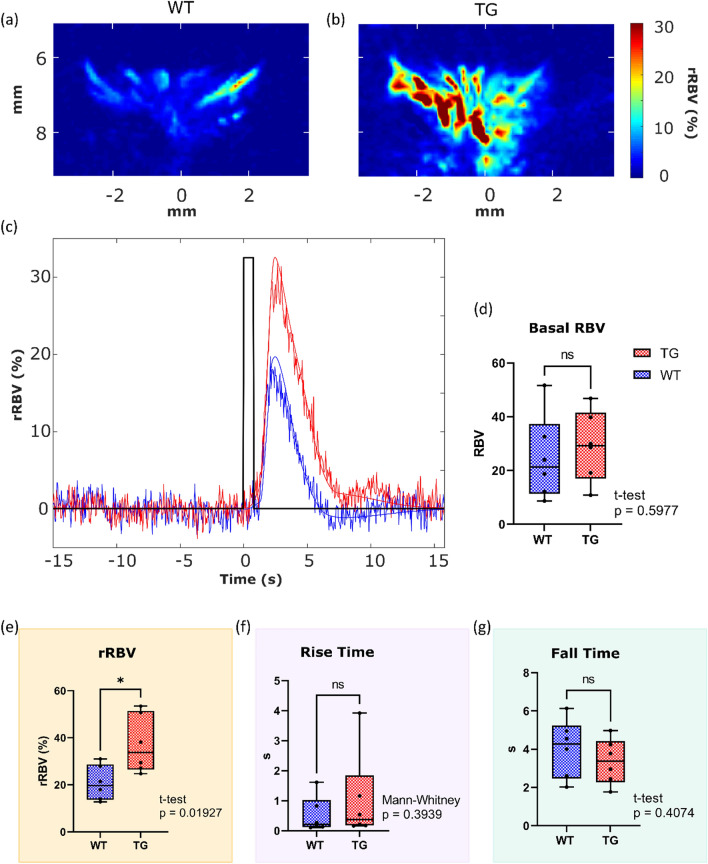
Changes in the eye neurovascular coupling in the TgF344-AD rat model. (**a**), (**b**) Example activation maps in a WT and in a TG animal. (**c**) Examples of trial-averaged eye neurovascular responses in a WT animal (Blue) and a TgF344-AD rat (red), illustrating the greater amplitude in the transgenic animal. (**d**)–(**g**) quantification of the different parameters of the eye neurovascular responses showing an increased response amplitude with an average of 20.83% for WT and 37.25%for TG (**e**). There was no significant difference in the basal blood volume (panel **d**), the rise, and the fall time (panels **f** and **g**).

We measured an increased response amplitude in TgF344-AD rats compared to control rats (*p* < 0.05), with a mean response value of 20.83 ± 3.03% in control and 37.25 ± 5.06% in TgF344-AD. No significant differences were found in the rise and fall times of the response between groups, as seen in Fig. 5.

## Discussion

The retina is an integral part of the central nervous system and is widely considered a natural window to the brain, and it stands to reason that some significant neurodegenerative pathologies could exhibit ocular manifestations. Interestingly, prospective studies have shown that retinal microvascular alterations could predict the risk of stroke^32^, Parkinson’s Disease, or Alzheimer’s Disease^33,35^.

Over the past 30 years, several optical imaging modalities have been developed to measure the optic nerve or retinal vascular response to changes in retinal neural activity. Arteriole dilation increases retinal blood velocity, which can be measured using techniques based on the Doppler effect. Bi-directional laser Doppler velocimetry yields an absolute measure of red blood cell velocity. Laser Doppler flowmetry measures relative red blood cell velocity, blood volume, and flux in the optic nerve head and choroid. Laser speckle flowmetry yields two-dimensional images of relative blood velocity on the retina surface. The Dynamic Vessel Analyzer (DVA) is another promising optical tool that measures indirect retinal vascular dilatation of superfical vessels in response to diffuse illuminance flicker and has been investigated in the context of Alzheimer’s disease^16,17^.

In this work, we investigated how retinal functional ultrasound imaging could be used to assess retinal function and neurovascular alterations in vivo*.*

We first demonstrated how ultrasound localization microscopy (ULM), using microbubbles, allows us to construct highly detailed maps of the microvasculature in vivo and to quantify hemodynamics parameters at a microscopic scale in depth. ULM can be used to image the main retinal arteries and their velocity, which could be used in clinics to monitor glaucoma as proposed in Qian et al.^36^, diagnose optic nerve compression^37^ or ophthalmic artery occlusion.

Without contrast agents, we measured the change in retinal blood volume during very short light stimulations (0.8 s) and could map the activated areas in the retinal bed with high confidence (z scores > 9). We measured consistent blood volume increase up to 30% compared to baseline, much stronger than the relative increase of diameter reported in DVA or BOLD signal change in fMRI.

We showed that 12 min would be sufficient to obtain very stable values (< 5% relative error) when estimating the main parameters of the hemodynamic response function. We used a non-linear fitting of the response to inferring the hemodynamic response function but also used short visual stimuli of 0.8 s to be as close as possible to the canonical response. We also choose a simple model for the hemodynamic response function with direct parameters, in which each independent parameters control parts of the function and thus avoids local minima. The parameters extracted here (amplitude, rise time, and fall time) could be helpful in early diagnosis as they may reflect the mechanisms behind the neurovascular coupling alterations and associated pericytes dysfunctions. We intend to improve the technique further so that more parameters can be reliably extracted, for instance, to better characterize the undershoot following the RBV increase. Another important step would be to study the effect of light intensity, duration, and flicker frequency to improve the measurements as it was done in optics^38^ or with fUS in the rat brain^39^.

Finally, as a proof of concept, we tested how the main hemodynamic response function parameters could discriminate between wild-type rats and TgF344-AD rats and found a significant increase in the hemodynamic response amplitude in the TgF344-AD group but no significant change in the basal RBV, rise, or fall times. This significantly increased response in TgF344-AD rats can be surprising as most studies postulate neurovascular alterations as a decreased response. For instance, investigating vascular reactivity in the brain of TGF-344-AD rats using two-photon fluorescence microscopy, Joo et al. report a relative decrease in vascular reactivity during hypercapnia^40^. However, sustained systemic hypercapnia may rely on different neurovascular mechanisms than short visual stimuli in the retina. In patients, several studies have investigated the hemodynamic response to light stimuli in the retina in the context of Alzheimer’s disease. Consistent with our findings, Kotliar et al.^16^ found a similar increase in the retina hemodynamic response to a light stimulus. In their study, they used DVA on subjects with Alzheimer’s disease dementia, subjects with mild cognitive impairment, and healthy subjects. Vessel dilatation was more extensive in mild cognitive impairment patients and even more so in subjects with Alzheimer’s than in healthy subjects. However, another recent study reports contradictory findings with lower responses observed in Alzheimer’s patients using the same DVA technique^17^. More fundamental studies will thus be needed to properly understand the mechanisms at play and the differences between clinical observations and the TgF344-AD model used in the study. We hope that rfUS as a new translational technique will help gather more insight into such mechanisms. It would be interesting to evaluate the differences in HRFs between the same animal’s retina and brain. Preliminary results from our group show a similar increase in the *superior colliculus* between wild-type and TgF344-AD but will need to be further explored.

Although we did not try to follow the clinical guidelines from the Food and Drug Administration for this preclinical proof-of-concept study, the acoustic parameters used here were not very high and indicated a possible translation of the technique to clinics. Indeed, we estimated in this study the spatial peak temporal averaged intensity (Ispta) to be 59 mW/cm^2^ and the mechanical index (MI) to be 0.43. These values are not much higher than the safety regulations for clinical evaluation^41^, corresponding to a mechanical index limit of 0.23 and an acoustic intensity of 50mW/cm^2^ (much more stringent than the conventional limits MI = 0.9 and Ispta = 720 mW/cm^2^ required for all other organs). Retinal fUS imaging must thus be calibrated and slightly optimized to stay below those limitations in clinical settings.

Another limitation of our study is that the probe was manually positioned using vascular anatomical landmarks and could be off the main activation area. It could thus be helpful to improve positioning by exploiting visual fixation or using a 3D ultrasound acquisition device^42,43^ to image the whole retina at once. Finally, it is known that the crystalline lens has a different speed of sound^44^ than tissue (1640 m/s versus 1540 m/s) and could induce strong aberrations in reconstructed ultrasound images. It could thus further improve our images to also correct for the lens aberration^45^.

## Conclusion

We demonstrated for the first time functional ultrasound imaging in the retina in vivo. We assessed task-evoked neurovascular responses and obtained robust activation maps with hemodynamic change to light stimulation up to 30% in the rat retina. We could discriminate between wild-type and Alzheimer’s disease model rats based on hemodynamic response parameters with a significant increase of the evoked response in the transgenic rats. The high sensitivity of rfUS to monitor blood volume during activation could detect early neurovascular alterations in pathologies such as Alzheimer’s disease or vascular dementia in its earliest stages before irreversible brain damage or cognitive decline has occurred. It could provide an economic tool for early, pre-symptomatic screening of a larger population than allowed by more expensive approaches (MRI or positron emission tomography). These techniques could, however, be used subsequently in an at-risk population to refine the diagnosis. rfUS could also be a valuable tool for drug development and treatment monitoring in preclinical and clinical settings.

## Supplementary Information

Supplementary Figure S1.

## Data Availability

Data supporting the findings of this study are available in the framework of an official collaboration between academic institutions.
