# Supplementary material for: Retinal functional ultrasound imaging (rfUS) for assessing neurovascular alterations: a pilot study on a rat model of dementia
Source: Sci Rep. 2022 Nov 14;12:19515. doi: 10.1038/s41598-022-23366-8 (PMC9663720; doi:10.1038/s41598-022-23366-8)
Supplement: Supplementary file 1 — Supplementary Figure S1. [file 41598_2022_23366_MOESM1_ESM.docx]

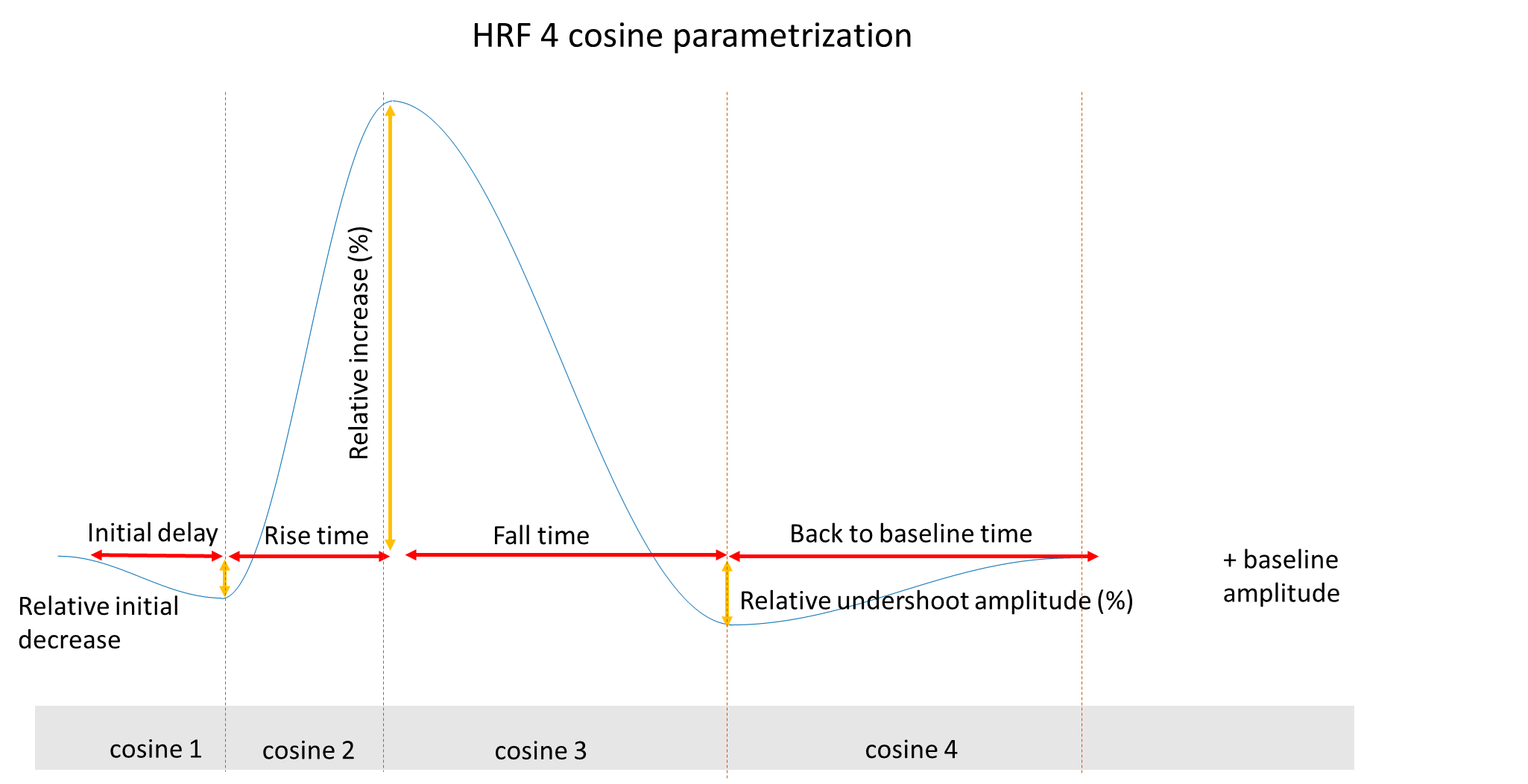


**Supplementary figure 1. Illustration of the 4 cosine HRF model.** The 4 cosine HRF model is a concatenation of 4 half cosine with different scale, sign and origin. It allows to model the hemodynamic response function initial decrease, increase, fall and undershoot as well as the baseline with 8 independent parameters. Each parameter has a distinct and independent effect on the HRF shape which allows direct interpretation and avoid local minimum solution during the optimization process.
